# A novel mutation in *ext2* caused hereditary multiple exostoses through reducing the synthesis of heparan sulfate

**DOI:** 10.1590/1678-4685-GMB-2020-0334

**Published:** 2021-05-21

**Authors:** Caixia Xian, Mingwei Zhu, Tianying Nong, Yiqiang Li, Xingmei Xie, Xia Li, Jiangui Li, Jingchun Li, Jianping Wu, Weizhe Shi, Ping Wei, Hongwen Xu, Ya-ping Tang

**Affiliations:** 1 Guangzhou Medical University, Guangzhou Women and Children’s Medical Center, Guangzhou Institute of Pediatrics, Guangzhou, Guangdong Province, P.R. China.; 2 Guangzhou Medical University, Guangzhou Women and Children’s Medical Center, Department of Pediatric Orthopedics, Guangzhou, Guangdong Province, P.R. China.

**Keywords:** Osteochondroma, hereditary, EXT1, EXT2, heparan sulfate

## Abstract

Hereditary multiple exostoses (HME) is a rare skeletal disorder characterized by the formation of multiple benign cartilage-capped tumors, usually in the metaphyseal region of the long bones. Over 70% of HME cases arise from monoallelic mutations in either of the two genes encoding the heparan sulfate (HS) synthesis enzymes, *ext1* and *ext2*. To identify more HME-associated mutations, genomic DNA from members of five independent consanguineous families with HME was sequenced with whole exome sequencing (WES). A novel heterozygous splice site mutation (c.1173+2T>A) in *ext2* was detected in all three affected members of family V. Further study showed that the novel mutation caused exon 7 of *ext2* mRNA to be skipped during splicing and caused a frameshift after the codon for Arg360, which results in the appearance of new 43 codons, followed by a termination codon. Although the resulting truncated protein was still localized to the Golgi, similar to the full-length EXT2, its HS synthesis activity decreased by 40%. In this study, a novel splice site mutation in *ext2* was identified and suggested to be a pathogenic mutation of HME, which may expand the genetic etiology spectrum of HME and may be helpful for clinical genetic counseling and prenatal diagnosis.

## Introduction

Hereditary multiple exostoses (HME), also known as hereditary multiple osteochondroma, is a rare genetic skeletal disorder characterized by the formation of cartilage-capped benign bone tumors, usually in the metaphyseal region of the long tubular bones, such as the bones of limbs, shoulder blades, ribs and pelvis, with symmetrical distribution (Stieber and Dormans, 2005). These growing osteophytes can compress the nearby soft tissues, causing pain and limited joint movement. HME also manifested with short stature, limb-length discrepancies, forearm deformities and valgus of the knee and ankle (Wicklund et al., 1995). The estimated prevalence of HME is approximately 0.02‰. In addition, up to 2% of cases could develop into malignant chondrosarcoma or osteosarcoma (Guo et al., 2019).

Over 70% of HME cases were caused by the heterozygous mutation of genes encoding exostosin-1 (EXT1) or exostosin-2 (EXT2), which map to chromosome 8q 24.11-q24.13 and 11p12-p11, respectively (Cook et al., 1993; Wu YQ et al., 1994; Bovée, 2008). Of these identified pathogenic variants of HME, 80% are nonsense, frameshift, or splicing mutations, which typically cause premature termination of translation (Wuyts and Van Hul, 2000). Both EXT1 and EXT2 are endoplasmic reticulum type II transmembrane glycoproteins. In the biosynthesis of heparan sulfate (HS), EXT1 and EXT2 form HS copolymerase in the Golgi to catalyze the alternating addition of D-glucuronic acid and N-acetylglucosamine residues to elongate the HS glycosaminoglycan chain (McCormick et al., 2000; Esko and Selleck, 2002). The attachment of HS to some cell-surface or extracellular proteins makes them heparan sulfate proteoglycans (HSPGs). HSPGs play important roles in multiple signaling pathways via their HS chains to regulate the distribution of morphogens or to modulate the interaction between extracellular ligands and their receptors (Li and Kusche-Gullberg, 2016). In patients with HME, HS levels were significantly reduced due to heterozygous loss-of-function mutations in the *ext1* or *ext2* genes, which altered the activity of multiple signaling pathways involved in chondrocyte differentiation and skeletogenesis, such as the FGF, BMP and IHH signaling pathways (Reddi, 2001; Lai and Mitchell, 2005; Reijnders et al., 2010). Recently, it was reported that low levels of HS increase the availability of BMP ligands and BMP receptor dynamics to enhance the activity of the BMP pathway. A pathogenesis model of HME has been proposed, suggesting that ectopic BMP signaling in progenitor cells in the perichondrium mediates osteochondromagenesis (Jiao et al., 2007; Cuellar and Reddi, 2013; Inubushi et al., 2017; Pacifici, 2018).

In the present study, we genetically analyzed five independent consanguineous families with HME (family I-V). In family V, a three-generation family with three affected individuals, a novel heterozygous splice site mutation in *ext2*, c.1173+2T>A, was identified via WES and validated by Sanger sequencing. Further study showed that the novel mutation caused exon 7 of *ext2* mRNA to be skipped while splicing and elicited a frameshift after the codon for Arg360, which results in appearance of new 43 codons, followed by a termination codon. Although the resulting truncated protein could normally localize to the Golgi, its HS synthesis activity was severely disrupted, which confirmed that the c.1173+2T>A in *ext2* is a novel pathogenic mutation of HME. Our findings expanded the genetic etiology spectrum of HME and may be helpful for clinical genetic counseling and prenatal diagnosis.

## Subjects and Methods

### Subjects

**Figure 1 -:**
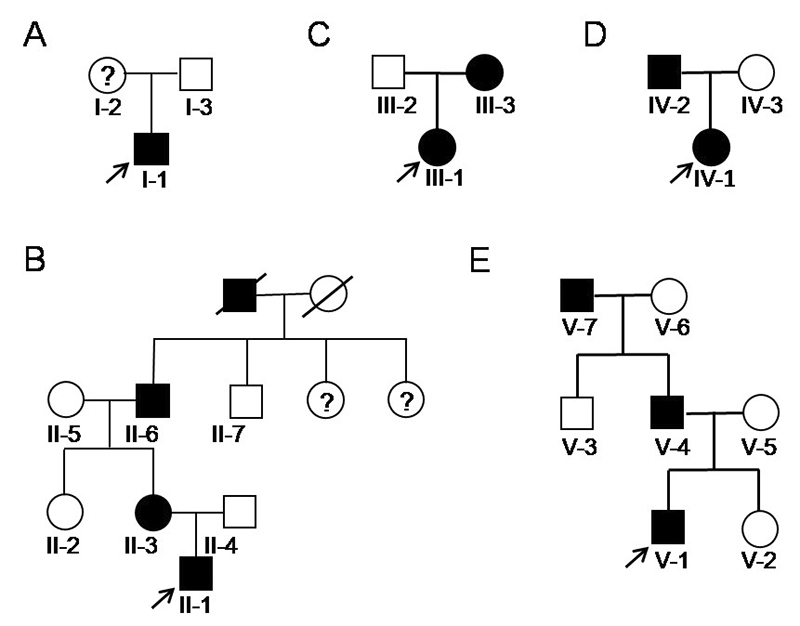
Pedigree of five families with HME. **A**: family I; **B**: family II; **C**: family III; **D**: family IV; **E**: family V. Arrow indicates the proband of each family. Question marks inside a circle or square indicated suspected patients. An oblique line means that the individual is decreased.

HME patients from five different consanguineous families were identified in the Guangzhou Women and Children’s Medical Center (Figure 1). Clinical and radiographic examinations were performed. The diagnosis was based on the existence of two or more exostoses at the juxta-epiphysial regions of long bones (Peterson, 1989). Available clinical data are shown in Table 1. This project was approved by the Human Ethics Committee of the Guangzhou Women and Children’s Medical Center. All subjects or their legal guardians signed informed consent forms.

**Table 1 -:** Clinical data of HME patients.

Subject	Proband sex	Age of onset	Localization
I-1	Female	3 years	Right ulna
II-1	Male	4 years	Bilateral distal femur, proximal tibia and fibula, distal tibia, left scapula
III-1	Male	1 years	Right tibia, right ulna
IV-1	Male	2 years	Left ulna, ribs
V-1	Female	4 years	Thoracic cage, right index finger, right seventh rib, right ulna

### Whole exome sequencing

Genomic DNA (gDNA) was extracted from the peripheral blood of all subjects. The concentration of gDNA was determined by a Qubit Fluorometer. The integrity and purity were detected with agarose gel electrophoresis. Then, WES was performed at the Beijing Genomics Institute (Shenzhen, China). gDNA was randomly fragmented to an average size of ~350 bp and subjected to DNA library construction using established Illumina paired-end protocols. The qualified libraries were sequenced with the Illumina HiSeqXten System to generate 150bp paired-end reads. The raw data were collected using Illumina Base Calling software (bcl2fastq) and sent to Genergy Bio (Shanghai, China) for analysis. The human genome assembly hg19 (GRCh37) was used as the reference sequence. The Genome Analysis Toolkit (GATK v3.3.0) was employed to detect single nucleotide variants and indels (SNV/INDEL). ANNOVAR software was used to annotate these variants. All variants were further filtered according to the type of mutations, the mode of inheritance, the frequency of mutations, and the pathogenicity of mutations. The pathogenicity of the identified missense variants was assessed *in silico* using SIFT, Polyphen2, and Mutation Taster software.

### Sanger sequencing

gDNA extracted from peripheral blood was used as a template. The primer sets were designed according to the position of each mutation (Table S1). The region of the *ext1* or *ext2* gene, encompassing the mutation site, was amplified by PCR. The qualified products were sent to Shanghai Sangon Biotech (Shanghai, China) for sequencing.

### *In silico* analysis

Two online tools were used in the present study. CRYP-SKIP (http://cryp-skip.img.cas.cz/) was used to estimate the probability of cryptic splice site activation and exon skipping of the splice site mutation. Human Splicing Finder (HSF) (http://www.umd.be/HSF3/) was used to predict its effects.

### Analysis of *EXT2* mRNA

Total RNA was extracted from the peripheral blood using TRIzol (Ambion, USA) with a standard procedure. To synthesize cDNA, 1 μg of total RNA was reverse transcribed with the RevertAid First Strand cDNA Synthesis Kit (Thermo Fisher Scientific, USA). The cDNA products were used as templates for PCR amplification. Three primer sets, Pair-1, Pair-2, and Pair-3, were used (Table S1). The PCR products were resolved on a 2% agarose gel. For TA cloning, the PCR products amplified with Pair-1 using the cDNA of the proband and control as templates were cloned into the pGEM-T Easy vector (Promega, USA). Then, *E. coli* DH5α cells were transformed with these vectors. The bacteria were cultured on ampicillin/X-gal/IPTG agar plates. Thirty positive clones were randomly selected for the proband and 14 for the control. Sequencing was conducted at Shanghai Sangon Biotech (Shanghai, China).

To determine the relative expression levels of *ext2* mRNA, RT-qPCR was performed. cDNA samples from the proband and control were amplified using SYBRPremixExTaq II (Takara Biotechnology) and detected using the two-step Real-Time PCR System (Thermo Fisher Scientific, USA). The primer set (forward 5-’AGGACCTAGAAGCCCTCCAG-3’, reverse 5’-GCCAGCTTGTAACACATCGC -3’) upstream of the mutation site (c.1173+2T>A) was used to amplify both the wild-type and mutant transcripts. The expression of the housekeeping gene GAPDH in each sample was used as an internal control. The data analysis was performed with the ΔΔC_T_ method.

### Construction of expression plasmids and transfection of HEK293 cells

The cDNA corresponding to the full-length and mutant *ext2* open reading frames (EXT2-FL and EXT2-DEL) was amplified from cDNA libraries that were constructed with total RNA extracted from the peripheral blood of the control and proband, respectively. The following primers were used: forward 5-’GTCGACATG TGTGCGTCGGTCAAGTA-3’; reverse 5’-TCATAAGCTGCCAATGTTGGGGAA GC-3’. The amplified product was cloned into the pGEM-T Easy vector and subsequently sequenced. Meanwhile, the pBudCE4.1-EGFP expression vector was modified to obtain the pBudCE4.1-EGFP-N-myc construct, which expresses an N terminal-myc tag under the CMV promoter. Then, EXT2-FL and EXT2-DEL cDNAs were inserted into pBudCE4.1-EGFP-N-myc to generate the two constructs, N-myc-EXT2-FL and N-myc-EXT2-DEL, respectively. To express EXT2-FL or EXT2-DEL together with EXT1, the cDNA corresponding to the full-length *ext1* open reading frame was amplified from a commercial human EXT1 ORF clone (Youbao Biological, China) and subcloned into pBudCE4.1 to generate the pBudCE4.1-EXT1 construct. Then, EXT2-FL and EXT2-DEL cDNAs were inserted into pBudCE4.1-EXT1 to generate the two constructs, EXT2-FL/EXT1 and EXT2-DEL/EXT1, respectively.

These constructs were transiently transfected into HEK293 cells using Lipofectamine 2000 (Invitrogen, USA). HEK293 cells were cultured in Dulbecco’s Modified Eagle’s medium (DMEM) supplemented with 10% (v/v) fetal bovine serum (Gibco), 1% penicillin G-streptomycin, and zeocin at a concentration of 200μg/ml.

### Western blotting

Following transfection for 48 h, HEK293 cells were harvested and lysed on ice in RIPA buffer containing protease inhibitor cocktail for 30 min. The protein concentration was determined using a Pierce BCA Protein Assay kit (Thermo Fisher Scientific, USA). An equal amount of protein from each sample was separated on a 7.5% SDS-PAGE, and then transferred to PVDF membrane for immunoblot analysis. The following primary antibodies were used: mouse anti-myc (1:1000, Cell Signaling Technology) and mouse anti-GAPDH (1:1000, Cell Signaling Technology). HRP-conjugated anti-mouse antibody was used as secondary antibody.

### Immunofluorescence and confocal imaging

HEK293 cells were transiently transfected with the corresponding constructs on coverslips. Forty-eight hours after transfection, the cells were rinsed three times with PBS, fixed with 4% paraformaldehyde in PBS for 15 min, permeabilized in 0.05% Triton X-100 for 15 min, and blocked with 5% normal goat serum in PBS for 1 h at room temperature. The cells were then incubated with mouse anti-myc (1:1000, Cell Signaling Technology) and rabbit anti-GM130 (1:2500, Sigma) at 4˚C overnight. Then, the cells were rinsed three times with PBS and incubated with Alexa Fluor 647 goat anti-rabbit IgG (1:1000) and Alexa Fluor 549 goat anti-mouse IgG (1:1000) for 2 h. Finally, the stained cells were washed three times with PBS and mounted in Vectashield containing DAPI (Vector Lab). Confocal images were captured on a Leica TCS SP8 laser scanning confocal microscope. Images shown in the same figure were acquired using the same gain from samples that had been simultaneously fixed and stained.

### Enzyme linked immunosorbent assay (ELISA)

The levels of HSPG-related proteins were quantified in treated HEK293 cells by ELISA assays using an ELISA Kit for Heparan Sulfate Proteoglycans (USCN Life Science, USA) in accordance with the manufacturer’s instructions.

### Statistical analysis

An unpaired Student’s *t*-test was used to evaluate the significance of the experiments. *P*-values<0.05 were considered to be significant.

## Results

### Mutation screening

**Figure 2 -:**
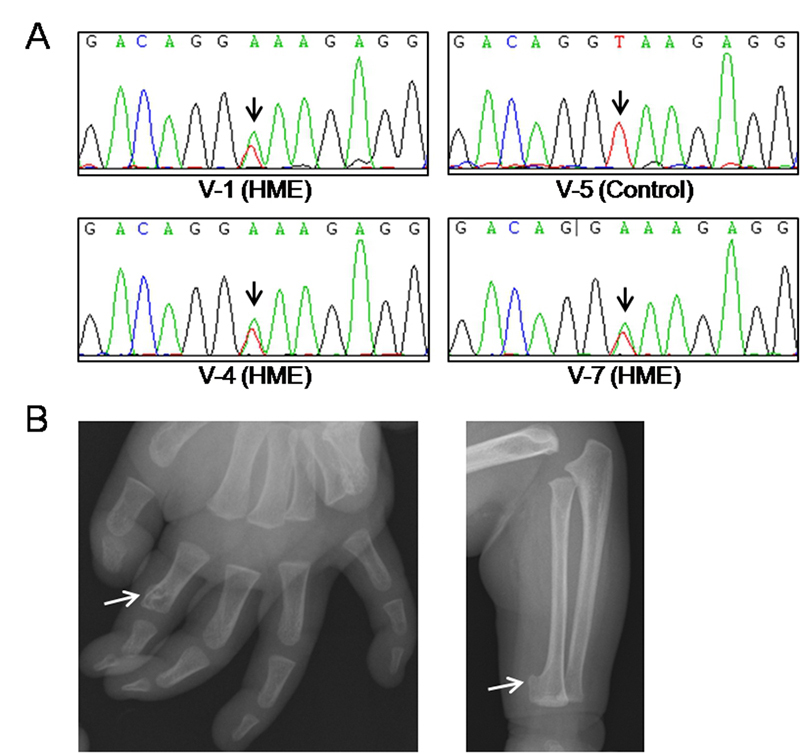
Identification of a novel mutation in *ext2* gene cosegregated with HME. A. Sanger sequencing results from genomic DNA of the members of family V. All HME patients, V-1, V-4 and V-7, carry the heterozygous mutation of *ext2* gene, c.1173+2T>A*.* The unaffected member is as control. The black arrows indicate the point of mutation. B. X-radiographic examination of the proband of Family V. Osteochondroma was indicated in the distal end of the right index finger, distal end of the right ulnar by white arrows.

To identify more HME-associated mutations, we recruited five independent consanguineous HME families. gDNAs from patients and their relatives were sequenced with whole exome sequencing. As shown in Table 2, two nonsense mutations were identified in the *ext1* gene in families I and III. One nonsense mutation and one intronic mutation were identified in the *ext2* gene in families IV and V, respectively. In family II, we did not detect any alteration in the *ext1* or *ext2* gene. All these mutations were heterozygous. Finally, we confirmed these alterations with Sanger sequencing (Figure 2A, and data not shown).

**Table 2 -:** Mutations identified in *ext1* and *ext2* gene from HME families.

Family	Gene	cDNA change^a^	Protein change	Status
I	*ext1*	c.1776C>G	p.Tyr592X	Novel
II		No mutations detected		
III	*ext1*	c.600G>A	p.Try200X	Recurrent
IV	*ext2*	c.1286G>A	p.Try429X	Recurrent
V	*ext2*	c.1173+2T>A	p.Arg360fs43X	Novel

^a^ The adenosine of the start codon is assigned nucleotide position +1.

### c.1173+2T>A, a novel HME-associated mutation in *ext2*

Next, we focused on the intronic mutation c.1173+2T>A in the *ext2* gene*,* which was not reported previously. In family V, this mutation cosegregated with disease phenotypes and was not detected in unaffected members (Figure 1E and Figure 2A). The proband (V-1) was diagnosed with HME at age 4. With regional X-radiographic examination, multiple exostoses were detected at his thoracic cage, right index finger, right seventh rib and right ulnar (Figure 2B). All other affected members of this family also have multiple exostoses at the metaphyses of the long tubular bones, which was confirmed by physical examination.

### c.1173+2T>A mutation results in aberrant *ext2* splicing transcript

**Figure 3 -:**
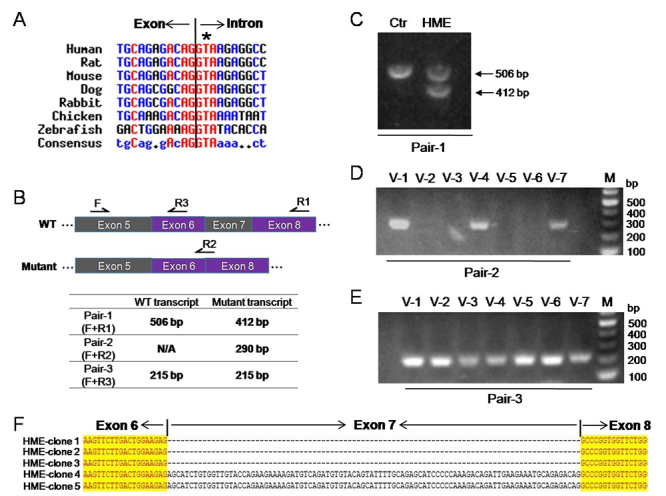
Aberrant splicing transcripts of *ext2* with exon7 being skipped. A. Alignment of *ext2* homologous gene sequences from 7 species. T residue (indicated by star mark) at the second position of intron 7 is included in a highly conserved splicing donor site. B. The position of primers designed to distinguish the two potential outcomes, exon 7 skipping and cryptic splice site activation in exon 7, was shown. The table showed the predicting length of the PCR products amplified by the three pair of primers in the case of exon 7 skipping. C, D, E. The agarose gel electrophoresis of the PCR products. F. TA cloning and sequencing results of aberrant splicing transcripts of *ext2*. Three of the five randomly picked clones showed exon 7 skipping.

The c.1173+2T>A mutation localizes at the +2 position of intron 7, which is involved in a splicing donor site. The T residue at this position was highly conserved across different species (Figure 3A). To predict the molecular consequence of the mutation, CRYP-SKIP analysis was performed, which demonstrated that the mutation most likely led to exon 7 skipping. The probability of exon 7 skipping (1-P_CR-E_) was 0.63, while cryptic splice site activation in exon 7 (P_CR-E_) was 0.37 (Figure S1).

Then, we extracted total mRNA from peripheral blood and designed three pairs of primers to distinguish these two potential outcomes by RT-PCR using agarose gel electrophoresis (Figure 3B). With Pair-1 primers, we amplified the wild-type transcript in both the proband and his mother (control), while a shorter mRNA variant was amplified only in the proband (Figure 3C). In Pair-2 primers, the reverse primer was designed to span exons 6 and 8 to detect the abnormally spliced transcript. As expected, Pair-2 primers functioned only in the amplification of HME patient samples (Figure 3D). These data strongly supported that aberrant *ext2* splicing transcripts without exon 7 only existed in HME patients, not in normal individuals. Finally, we performed TA cloning and sequencing of the PCR product with Pair-1 primers. In the proband, both wild-type and aberrant transcripts with exon 7 skipping were detected. However, in the control, only wild-type *ext2* transcripts were identified (Figure 3F). In addition, no splicing transcripts with cryptic splice sites were identified even we randomly sequenced 30 TA clones in the proband (Table S2). The results further confirmed that the c.1173+2T>A mutation caused exon 7 skipping in the *ext2* gene.

### Molecular consequence of abnormal splicing in *ext2*: mRNA degradation and protein truncation

**Figure 4 -:**
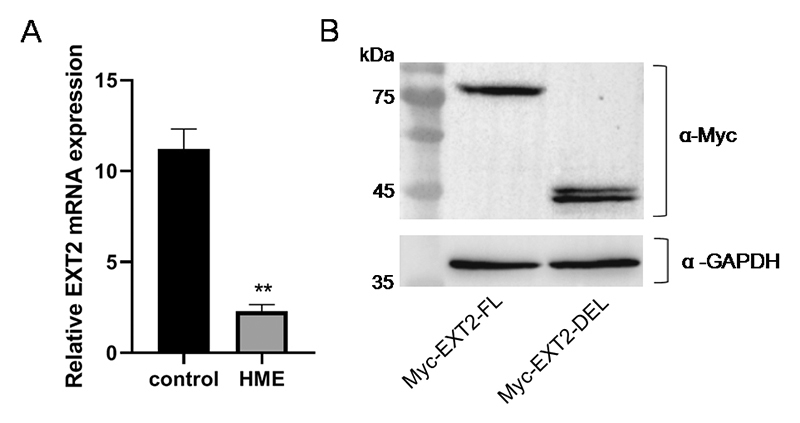
Molecular consequence of the abnormal splicing in *ext2* transcripts. A. Reverse transcription-quantitative polymerase chain reaction analysis of the expression levels of *ext2* mRNA in HME patient and control (n=4, *P*< 0.001). B. Western blot of recombinant N terminal-myc tagged full-length and mutant EXT2 protein expressed in HEK293 cells.

The *ext2* gene contains 14 exons. The encoded protein, consisting of 718 amino acids, mainly includes three domains: the transmembrane domain, exostosis and glycosyl transferase family 64 from the N- to C-terminus. To assess the possible effect of the alternative transcript, the mRNA and protein variants of EXT2 were further investigated. First, we quantified the relative level of *ext2* mRNA isolated from peripheral blood. As shown in Figure 4A, the levels of *ext2* mRNA in the proband containing wild-type and mutant transcripts were significantly lower than those in the control, which indicated that the mutant transcripts were unstable and degraded.

According to our analysis, the excision of exon 7 in *ext2* mRNA would lead to a frameshift after the codon for Arg360 which results in appearance of new 43 codons, followed by a termination codon. To verify this prediction, we constructed two vectors, N-myc-EXT2-FL and N-myc-EXT 2-DEL, which expressed N terminal-myc, tagged full length EXT2 and mutant EXT2, respectively. As expected, in HEK293 cells, N-myc-EXT2-FL expressed full-length EXT2 with a molecular weight of approximately 75 kDa. In cells transfected with N-myc-EXT2-DEL, only shorter peptides with molecular weights of approximately 45 kDa were detected, consistent with the prediction (Figure 4B). These results suggest that the abnormal splicing of *ext2* causes not only the degradation of mRNA but also the truncation of protein.

### Truncated EXT2 showed reduced activity of HS synthesis

**Figure 5 -:**
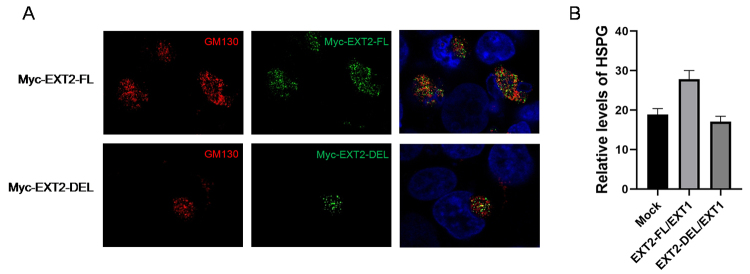
The truncated EXT2 protein has normal localization, but reduced activity of HS synthesis. A. Subcellular localization of myc-EXT2-DEL and myc-EXT2-FL (green) in HEK293 cells. Golgi was indicated by GM130 (red). Nucleus was indicated by DAPI (blue). B. Statistical analysis of the relative expression level of HSPGs synthesized by HEK293 cells transfected with the empty vector (Mock) or the EXT constructs as indicated. EXT2-FL/EXT1 *vs.* Mock, *P*=0.042; EXT2-DEL/EXT1 *vs.* EXT2-FL/EXT1, *P*=0.028.

In the biosynthesis of HS, EXT2 and EXT1 form HS copolymerase in the Golgi to catalyze the alternating addition of D-glucuronic acid and N-acetylglucosamine residues to elongate the HS glycosaminoglycan chain. To investigate the cellular function of truncated EXT2, its subcellular localization was studied first. Cellular immunofluorescence revealed that myc-EXT2-DEL was located in the Golgi apparatus with no notable changes compared with myc-EXT2-FL (Figure 5A). Next, we determined the HS synthesis activity of EXT2-DEL. Two vectors, EXT2-FL/EXT1 and EXT2-DEL/EXT1, were transfected into HEK293 cells. ELISA showed that the exogenous EXT2-FL/EXT1 significantly increased the synthesis of HS, while EXT2-DEL/EXT1 failed (Figure 5B). These results indicated that the activity of HS synthesis of truncated EXT2 decreased dramatically compared with full-length EXT2, even with normal localization.

## Discussion

HME is a rare dominantly inherited skeletal disorder characterized by the formation of cartilage-capped benign bone tumors, usually in the metaphyseal region of the long tubular bones. Over 70% of HME cases are caused by heterozygous mutations of the *ext1* or *ext2* gene. In the present study, to identify more HME-associated mutations, we genetically analyzed five independent consanguineous families with HME. With whole exome sequencing, two nonsense mutations in *ext1* (p.W200* and p.Y592*) and one nonsense mutation (p.W429*) and one intronic mutation in *ext2* (c.1173+2T>A) were detected*.* The c.1173+2T>A mutation was not reported previously and cosegregated with HME patients in family V, which suggested that this mutation may cause HME.

Splice site mutations can induce the following effects on splicing: exon skipping and activation of a cryptic splice site (Divina et al., 2009; Barash et al., 2010). The c.1173+2T>A mutation was located at the 5’ splice site of intron 7 of the *ext2* gene, which was predicted to cause exon 7 skipping through *in silico* analysis. RT-PCR and sequencing of TA clones revealed that the alternative transcripts of *ext2* without exon 7 can only be detected in the proband and other affected members of family V, not in normal individuals, which strongly supported the prediction. Furthermore, two similar mutations, c.1173+1G>T and c.1173+1G>A, which are also involved in the 5’ splice site of intron 7 of *ext2*, have been reported to cause the excision of exon 7 (Wuyts et al., 1998; Francannet et al., 2001; Lonie et al., 2006; Yang et al., 2010; Wu Y *et al.*, 2013). Thus, we concluded that the novel mutation c.1173+2T>A identified in our study led to abnormal splicing and exon 7 skipping in the *ext2* gene.

Although the excision of exon 7 in *ext2* transcripts was reported in two prior studies, we demonstrated for the first time the molecular and cellular consequences of abnormal splicing in the present study. The expression level of EXT2 was determined with RT-qPCR. The results showed that the relative levels of *ext2* mRNA, containing wild-type and mutant transcripts, were significantly lower in patients than in normal individuals, which indicated that the mutant transcripts were unstable and degraded. Previous reports predicted that the loss of exon 7 would lead to a shift in the codon-reading frame at Arg360 followed by the synthesis of 43 novel amino acids that terminate with a stop codon at position 404. However, no evidence was provided. We cloned the wild-type and mutant transcripts and expressed them in HEK293 cells. In contrast to the full-length EXT2 expressed by the wild-type transcript, the mutant transcript expressed the truncated protein with a molecular weight of approximately 45 kDa, which matched the predicted size. The subcellular localization and molecular function of truncated EXT2 were further investigated. Similar to the full-length EXT2, the truncated EXT2 colocalized with GM130, which was used to indicate the Golgi apparatus, suggesting its normal subcellular localization. However, the HS synthesis activity of the truncated EXT2 was dramatically disrupted, as shown by the ELISA assay. As truncated EXT2 retains the transmembrane and exostosis domains, together with these results, it suggested that the transmembrane and exostosis domain determine the localization of EXT2, while the glycosyl transferase family 64 domainis involved in the synthesis of HS.

EXT2 and EXT1 proteins form a hetero-oligomeric complex in the Golgi apparatus to function as a glycosyltransferase in the polymerization of HS. The attachment of HS to some cell-surface or extracellular proteins converts them into heparan sulfate proteoglycans (HSPGs). HSPGs play important roles in the distribution and receptor binding of signaling molecules, such as transforming growth factor-β, BMPs, fibroblast growth factor, and Indian hedgehog, thereby regulating chondrocyte proliferation and differentiation (Cuellar and Reddi, 2013). Our findings showed that the c.1173+2T>A mutation caused the low expression level and reduction of HS synthesis activity of EXT2 and therefore the dramatic decrease of HS in HME patients. Recently, it was reported that low levels of HS increase the availability of BMP ligands and BMP receptor dynamics to enhance the activity of the BMP pathway in progenitor cells in the perichondrium, thereby inducing osteochondromagenesis.

In the present study, we genetically screened five independent HME families and identified a novel splice site mutation (c.1173+2T>A) in the *ext2* gene that cosegregated with the HME phenotype in one of these families. The mutation caused an abnormal transcript, with exon 7 being skipped, which led to the decreased expression of EXT2 mRNA in HME patients. In addition, we demonstrated for the first time the molecular and cellular consequences of the excision of exon 7. In HEK293 cells, although the truncated EXT2 translated from the abnormal transcript normally localized to the Golgi apparatus, its HS synthesis activity was notably reduced due to the absence of the glycosyl transferase family 64 domain. In conclusion, the novel c.1173+2T>A mutation caused the low expression and HS synthesis activity of EXT2 and therefore the decrease in HS levels, which underlies the pathogenesis of HME in the pedigree investigated in this study. Our findings may be helpful for genetic diagnosis and may also help to elucidate the pathogenesis of HME.

## Supplementary material

The following online material is available for this article:

Table S1 - Primers used to amplify and sequence the *ext1* and *ext2* gene.

Table S2 - TA clone and sequencing results of the proband of HME and normal control.

Figure S1 - Screenshot of the CRYP-SKIP output.
