# Supplementary material for: A novel mutation in ext2 caused hereditary multiple exostoses through reducing the synthesis of heparan sulfate
Source: Genet Mol Biol. 2021 May 21;44(2):e20200334. doi: 10.1590/1678-4685-GMB-2020-0334 (PMC8156126; doi:10.1590/1678-4685-GMB-2020-0334)
Supplement: Table S1 - [file 1415-4757-GMB-44-2-e20200334-s1.pdf]

Genetics and Molecular Biology
https://doi.org/10.1590/1678-4685-GMB-2020-0334

Supplementary Material to "A novel mutation in *ext2* caused hereditary multiple exostoses through reducing the synthesis of heparan sulfate"

Table S1 - Primers used to amplify and sequence the *ext1* and *ext2* gene.

Gene	ID	Forward	Reverse
ext1	Family I	5-'TGGGGCAAAATGTCAAGCAG-3'	5-'TGCCAAGAGGTTTCACTGGT-3'
	Family III	5-'ACATTCTAGCGGCCATCGAG-3'	5-'TTTGCCAGTCTTTGCCATGC-3'
ext2	Family IV	5-'CAACTTCTGATGGCAGCTGG-3'	5-'AACAAGACCCATGCCTCCTT-3'
	Family V	5-'CAGCCAGTGAAGAAGGGAGG-3'	5-'ACCCTCCAAAGAGCTTCAGC-3'
	Pair-1	5-'AGGACCTAGAAGCCCTCCAG-3'	5'-CACAGCAGGAGGGTCATTCC-3'
	Pair-2	5-'AGGACCTAGAAGCCCTCCAG-3'	5'-ACCACCGGGCCTCTTCCAGTC-3'
	Pair-3	5-'AGGACCTAGAAGCCCTCCAG-3'	5'-GCCAGCTTGTAAC ACATCGC-3'
