# Supplementary material for: A novel mutation in ext2 caused hereditary multiple exostoses through reducing the synthesis of heparan sulfate
Source: Genet Mol Biol. 2021 May 21;44(2):e20200334. doi: 10.1590/1678-4685-GMB-2020-0334 (PMC8156126; doi:10.1590/1678-4685-GMB-2020-0334)
Supplement: Table S2 - [file 1415-4757-GMB-44-2-e20200334-s2.pdf]

Genetics and Molecular Biology
https://doi.org/10.1590/1678-4685-GMB-2020-0334

Supplementary Material to "A novel mutation in *ext2* caused hereditary multiple exostoses through reducing the synthesis of heparan sulfate"

Table S2 - TA clone and sequencing results of the proband of HME and normal control.

Subjects	Clones with skipped exon7	Clones without skipped exon7	Total number of valid clones	Clones with skipped exon7/total number of clones
HME (V-1)	20	10	30	0.667
Control (V-5)	0	14	14	0
