# Supplementary material for: A novel mutation in ext2 caused hereditary multiple exostoses through reducing the synthesis of heparan sulfate
Source: Genet Mol Biol. 2021 May 21;44(2):e20200334. doi: 10.1590/1678-4685-GMB-2020-0334 (PMC8156126; doi:10.1590/1678-4685-GMB-2020-0334)
Supplement: Figure S1 - [file 1415-4757-GMB-44-2-e20200334-s3.pdf]

Genetics and Molecular Biology
https://doi.org/10.1590/1678-4685-GMB-2020-0334

Supplementary Material to "A novel mutation in *ext2* caused hereditary multiple exostoses through reducing the synthesis of heparan sulfate"

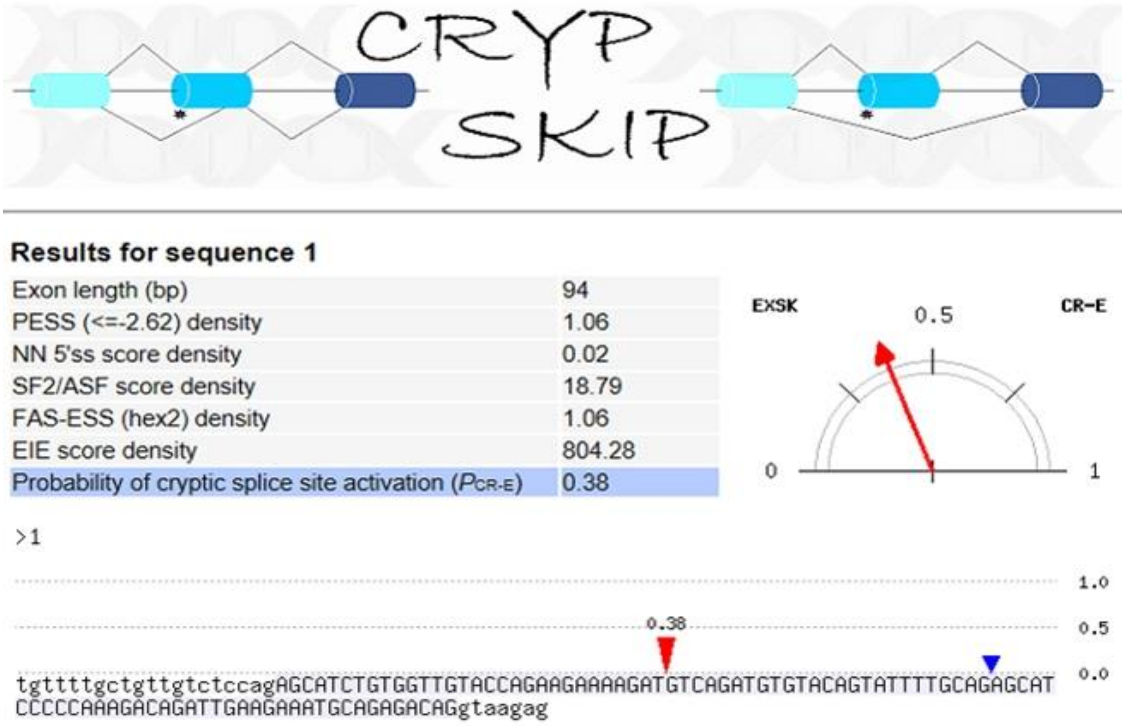

Figure S1 – Screenshot of the CRYP-SKIP output. The input *ext2* sequences included exon 7 (upper case letters) and the flanking intronic sequences (lower case letters). The list on the left showed the values used in the predictor and the data outputted. $P_{CR\text{-}E}$ was shown in light blue. The red arrow on the right pointed to EXSK in favor of exon skipping. The red vertical mark in the sequence indicates the predicted cryptic donor splice site. The blue vertical mark indicates the predicted acceptor site. PESS, putative exonic splicing silencers; NN 5'ss, neural network 5' splice sites; SF2/ASF, the most important SR protein for aberrant splice-site activation; FAS-ESS, ESSs discovered by a fluorescence-activated screen; EIE, exon and intron identity element.
